# Targeting tumor suppressor gene co‐mutations in *EGFR*‐mutant nonsmall cell lung cancer: How ACROSS2 sharpens the case for early intensification

**DOI:** 10.3322/caac.70072

**Published:** 2026-03-12

**Authors:** Krishna S. Gunturu, Hina Khan, Gilberto de Lima Lopes

**Affiliations:** ^1^ Hartford HealthCare Cancer Institute Hartford Connecticut USA; ^2^ The Warren Alpert Medical School of Brown University Providence Rhode Island USA; ^3^ Sylvester Comprehensive Cancer Center at the University of Miami and the Miller School of Medicine Miami Florida USA

The treatment landscape for *EGFR*‐mutated nonsmall cell lung cancer (NSCLC) continues to evolve with welcome speed. With the advent of third‐generation tyrosine kinase inhibitors (TKIs) like osimertinib, treatment has improved systemic and central nervous system control. However, patients who have NSCLC harboring concomitant tumor suppressor gene alterations, particularly *TP53* mutations, represent a biologically distinct subgroup with a poor prognosis and limited therapeutic options. TP53 co‐mutations in *EGFR*‐mutated NSCLC range from 40% to 68% in the real‐world setting. These *co‐mutated* tumors often behave aggressively, relapse earlier, and exhibit shorter lasting responses to targeted monotherapy.^1^, ^2^ In one retrospective study, 5753 patients with *EGFR*‐mutant NSCLC specimens were molecularly profiled.^3^ Patients younger than 50 years who had classical *EGFR* mutations had the longest median overall survival (OS) with osimertinib compared with both older adults with classical *EGFR* mutations (40 vs. 32 months; hazard ratio [HR], 0.692; *p* < .01) and older adults with atypical *EGFR* mutations (40 vs. 21 months; HR, 0.47; *p* < .0001), independent of *TP53* mutation status. However, compared with older adults (older than 50 years) with atypical *EGFR* mutations, older adults with classical mutations had longer OS only in the absence of concurrent *TP53* mutations (median OS, 39 vs. 28 months; HR, 0.735; *p* < .0001). In addition, *TP53* co‐mutations were enriched in the younger adult population (younger than 50 years; 76% vs. 62%, *q* < .05) compared with older adults.

One strategy to improve outcomes is upfront treatment intensification combining an *EGFR* TKI and chemotherapy. Contemporary randomized phase 3 trials, specifically FLAURA2 and MARIPOSA (ClinicalTrials.gov identifiers NCT04035486 and NCT04487080, respectively), have established that upfront treatment intensification improves disease control beyond what can be achieved with TKI monotherapy alone. In this evolving therapeutic landscape, the prospective ACROSS2 trial (ClinicalTrials.gov identifier NCT03400717) adds important confirmatory evidence. Rather than redefining standards of care, the trial reinforces an emerging consensus across studies: early treatment intensification is associated with improved outcomes in most patients, and no clearly delineated subgroup has been identified for whom initial monotherapy is preferred.

In this issue of *CA: A Cancer Journal for Clinicians*, investigators from the ACROSS2 phase 3 trial^4^ offer a compelling, thoughtfully designed attempt to improve outcomes in advanced *EGFR*‐mutant NSCLC with tumor suppressor gene co‐mutations. By selectively enrolling patients with *EGFR* exon 19 deletion or L858R NSCLC who also harbor tumor suppressor gene alterations—most commonly *TP53* but also *RB1*, *PTEN*, *APC*, and others — ACROSS2 is distinguished from broader, all‐comers combination trials like FLAURA2 and MARIPOSA.

This genomic enrichment strategy represents a biologically rational and clinically important question. The results are encouraging. Aumolertinib plus platinum–pemetrexed demonstrated a clinically meaningful improvement in progression‐free survival (PFS) compared with monotherapy (19.78 vs. 16.53 months; HR, 0.58; 95% confidence interval [CI], 0.34–0.97). These results demonstrated a significant PFS improvement (HR, 0.58) with an absolute gain of 3.3 months, a benefit similar in relative magnitude to that reported in the FLAURA2 trial (HR, 0.71) despite a more difficult‐to‐treat cancer. The authors' exploratory analysis examining *TP53* mutation domains and functional classifications provides additional insight into the heterogeneity within this molecularly defined subgroup. Although these analyses were limited by insufficient statistical power, they do establish a foundation for the design of future biomarker‐driven clinical trials.

## WHAT FLAURA2 AND MARIPOSA HAVE TAUGHT US

FLAURA2 demonstrated that combining osimertinib with platinum–pemetrexed chemotherapy significantly improves PFS and OS compared with osimertinib alone in an unselected *EGFR* exon 19 deletion or L858R population.^5^, ^6^ Importantly, FLAURA2 demonstrated that patients with TP53 alterations derived meaningful benefit from combination therapy; the median OS was 51.1 months with osimertinib plus chemotherapy versus 43.1 months with osimertinib alone (HR, 0.71; 95% CI, 0.40–1.27). Although the absolute benefit varied, the direction of the effect consistently favored combination therapy. We cautiously point out that *TP53* mutations were identified in 86 patients in the FLAURA2 trial.

Similarly, the MARIPOSA trial demonstrated superior PFS with amivantamab plus lazertinib compared with osimertinib monotherapy across all examined subgroups.^7^, ^8^ Patients with biologically adverse features, including TP53 alterations, experienced poorer absolute outcomes, but the relative benefit of intensification was preserved. The MARIPOSA trial has enrolled 293 patients with *TP53* status. The combination of amivantamab–lazertinib improved median PFS compared with osimertinib in patients who had *TP53* co‐mutations (18.2 vs. 12.9 months; HR, 0.65; 95% CI, 0.48–0.87; *p* = .003) Together, these trials argue against the notion that aggressive tumor biology should justify therapeutic de‐escalation and, rather, suggest a rationale for treatment intensification.

## WHERE ACROSS2 FITS

Against this backdrop, the phase 3 ACROSS2 trial should be interpreted as complimentary evidence supporting early intensification, rather than advocating molecular selection as a new standard of care. By prospectively enrolling patients with *EGFR*‐sensitizing mutations and concomitant tumor suppressor genes, ACROSS2 targets a population that has long been recognized to experience inferior outcomes with EGFR TKI monotherapy.^9^ The trial demonstrated a statistically significant improvement in PFS with aumolertinib plus platinum–pemetrexed compared with aumolertinib alone (HR, 0.58), with an absolute median gain of 3.3 months. Although numerically modest, this effect size is strikingly consistent with that observed in FLAURA2, despite enrichment for adverse biology in ACROSS2. Collectively, these findings reinforce a now well recognized pattern: combination therapy confers superior outcomes in genomically high‐risk disease. At the same time, ACROSS2 should not be construed as establishing a new standard of care for this population. Although the trial yields biologically plausible and clinically informative insights into tumor suppressor gene‐altered tumors, it delineates a subgroup in whom monotherapy is preferable, nor does it support molecular stratification as a rationale for withholding early treatment intensification. Indeed, the totality of evidence from broader studies, including FLAURA2 and MARIPOSA, argues against such a conclusion.

## IMPLICATIONS FOR CLINICAL PRACTICE

Taken together, the available evidence supports a pragmatic and increasingly clear approach to first‐line therapy in *EGFR*‐mutant NSCLC: combination‐based strategies—whether chemotherapy with *EGFR*‐TKI or dual‐targeted approaches—should be considered the preferred option for most patients with adequate performance status and those harboring co‐occurring tumor suppressor gene alterations. Single‐agent *EGFR*‐TKI therapy should be reserved for patients who are not candidates for an intensive regimen based on comorbidities and poor performance status.

Currently, molecular features such as TP53 or RB1 alterations should inform prognosis and counseling, but not routine de‐escalation decisions outside of clinical trials. ACROSS2 does not redefine the therapeutic paradigm; rather, it strengthens the one already established. Alongside FLAURA2 and MARIPOSA, it contributes to a growing and internally consistent body of evidence demonstrating that early intensification improves outcomes across the spectrum of *EGFR*‐mutant NSCLC, including in patients with genomically adverse disease. Notably, to our knowledge, this is the first study in which tumor suppressor gene alterations were studied in a prospective manner. Until robust evidence clearly identifies a subgroup that derives comparable benefit from monotherapy, combination treatment should remain the default strategy for patients who can tolerate intensification, with single‐agent therapy reserved for those in whom combination approaches are not feasible.

These results extend and strengthen exploratory analyses from prior studies suggesting that combination therapies, particularly chemotherapy in conjunction with TKI therapy, warrant consideration in patients with *EGFR* mutant lung cancer who harbor co‐occurring tumor suppressor gene alterations like *TP53*.

## CONFLICT OF INTEREST STATEMENT

Gilberto de Lima Lopes Jr reports research funding directly or through his institution from AstraZeneca, Lucence, Xilis, Bristol Myers Squibb Company, Merck Sharp & Dohme, EMD Serono, Blueprint Medicines, Tesaro, Bavarian Nordic, Novartis, G1 Therapeutics, AdaptImmune, GlaxoSmithKline, AbbVie, Rgenix, Pfizer, Roche/Genentech, Eli Lilly and Company, and Janssen; personal/consulting and/or advisory fees from Pfizer, AstraZeneca, Mirati Therapeutics, Coherus Biosciences, Regeneron, and Dr. Reddy’s Laboratories; honoraria from Boehringer Ingelheim, Blueprint Medicines, AstraZeneca, Merck, Janssen, Rigel, and Dr. Reddy’s Laboratories; stock or ownership interests in Lucence Diagnostics, Xilis, Biomab, Morphometrix, CDR‐Life, and Immorta Bio; and travel or accommodation support from multiple pharmaceutical companies outside the submitted work. Krishna S. Gunturu reports support for professional activities from Amgen, AstraZeneca, Bristol‐Myers Squibb Company, Daiichi Sankyo Company, and Merck & Company outside the submitted work. Hina Khan Reports personal/consulting fees from Boehringer Ingelheim, Daiichi Sankyo Company, Eli Lilly and Company, and Regeneron outside the submitted work.
